# Huntington’s disease- a case of early psychiatric symptoms and suicide

**DOI:** 10.1192/j.eurpsy.2021.1713

**Published:** 2021-08-13

**Authors:** A. Estalagem, H. Bastos, M.D.C. Cruz

**Affiliations:** 1 Psiquiatria E Saúde Mental, CHUA- Hospital de Portimão, Portimão, Portugal; 2 Psiquiatria, CHUA - Hospital de Portimão, Portimão, Portugal

**Keywords:** huntingtons, huntingtonsdisease, Depression, Suicide

## Abstract

**Introduction:**

Huntington’s disease is typically an inherited neurodegenerative disorder with autosomal dominant transmission. Early disease symptoms can include depression and behavioral changes, while physical and cognitive symptoms become evident later. Suicide and suicidal ideation are more frequent in these patients than on the general population. We present the case of a 50-year-old female patient with a history of depression and suicidal intents previous to her diagnosis. The patient committed suicide approximately 20 years after the beginning of her psychiatric symptoms.

**Objectives:**

To report a clinical case of early psychiatric symptoms and suicide in Huntington’s disease; To raise awareness for these comorbidities and for an adequate intervention in suicide prevention.

**Methods:**

The information was obtained by interviewing the patient and her family and by reviewing past medical reports. A brief literature review using the keywords “suicide”, “Huntington´s disease” and “psychiatric symptoms” was performed on PubMed.

**Results:**

The patient had a history of depression and five hospital admissions for suicidal intents during the ten years prior to the diagnosis. After the diagnosis and the beginning of physical symptoms, she maintained suicidal ideation until she committed suicide ten years later.

**Conclusions:**

This clinical case underlines the importance of being alert for early psychiatric symptoms of Huntington’s disease, especially if considering the patients’ probability of developing it. It also reinforces the need for suicidal ideation regular assessment and for pharmacological and non-pharmacological targeted therapy. Further investigation should be taken to understand which factors increase the risk for suicidal behavior and which moments during disease progression are crucial for prevention.

**Disclosure:**

No significant relationships.

